
JOURNAL INFORMATION
==============================
NLM Title Abbreviation: Wirtschaftsdienst
Journal ID: Wirtschaftsdienst
NLM Journal ID: 101086612

Wirtschaftsdienst (Hamburg, Germany : 1949)

ISSN: 0043-6275

ARTICLE INFORMATION
==============================
PMCID: PMC7584868
PMID: 33132452
DOI: 10.1007/s10273-020-2759-3
Article ID: 2759
Article version: 1
Subjects: Zeitgespräch

Industriestrategie der nächsten Generation für Deutschland

Kattel Rainer r.kattel@ucl.ac.uk

Mazzucato Mariana m.mazzucato@ucl.ac.uk

Haverkamp Keno keno.haverkamp@icloud.com

Ryan-Collins Josh j.ryan-collins@ucl.ac.uk

grid.83440.3b 0000000121901201 Institute for Innovation & Public Purpose (IIPP), University College London (UCL), 11 Montague Street, WC1B 5BP London, UK
Electronic publication date: 2020 Oct 24
Print publication date: 2020
Volume: 100
Issue: 10
First page: 757
Last page: 762
Copyright: © Der/die Autor(en) 2020
License: Dieser Artikel wird unter der Creative Commons Namensnennung 4.0 International Lizenz (https://creativecommons.org/licenses/by/4.0/deed.de) veröffentlicht.
Open Access wird durch die ZBW — Leibniz-Informationszentrum Wirtschaft gefördert.
License URL: https://creativecommons.org/licenses/by/4.0/

issue-copyright-statement: © The Author(s) 2020

==============================
Dieser Aufsatz basiert auf einer am 29. September 2020 auf der Tagung des Forum New Economy vorgestellten Studie „Challenge-driven economic policy: A new framework for Germany“ im Auftrag des Forum New Economy.

Deutschland tritt in eine neue Ära ein. Das übergreifende wirtschaftspolitische Konzept der vergangenen Jahrzehnte stützte sich vordergründig auf die ordoliberalen Prinzipien, die die Rolle des Staates darin sehen, dafür zu sorgen, dass die Rahmenbedingungen ein Funktionieren der Märkte ermöglichen. Nach dieser Auffassung sollte der Staat Marktversagen beheben und den Rest der Wirtschaft überlassen. Jüngste strategische Initiativen wie die Hightech-Strategie 2025, die Nationale Industriestrategie 2030 und die Kohleausstiegsstrategie versuchen jedoch, über die Marktfixierung hinauszugehen und die Grundlagen für einen aktiveren Staat zu schaffen. Wir können Veränderungen ordoliberaler Prinzipien in der erfolgreichen Energiewende-Politik der 2000er Jahre verfolgen. Mit dem Umgang mit COVID-19 hat Deutschland einen weiteren Schritt getan; es steht an der Spitze mutiger politischer Maßnahmen, um die Wirtschaft angesichts der Pandemie umzugestalten.

Innovations- und Industriepolitik dienen idealerweise zwei übergreifenden Zielen. *Erstens* der Generierung und Verbreitung von neuem Wissen und neuen Routinen. *Zweitens* der Lösung der größten sozioökonomischen Herausforderungen der Gesellschaft, d.h. der Mitgestaltung bei der Ausrichtung des Wachstums. Das deutsche Wirtschaftsmodell nach dem Zweiten Weltkrieg zeichnete sich durch eine diffusionsorientierte Politik aus, die für eine relativ stabile Dynamik der Wissensgenerierung und -verbreitung sowie für eine erhöhte internationale Wettbewerbsfähigkeit deutscher Unternehmen gesorgt hat. Im Schatten ihrer ordoliberalen Rhetorik setzte die deutsche Wirtschaftspolitik nach dem Zweiten Weltkrieg auf verdeckte Industrie- und Innovationspolitik und öffentlich-private Partnerschaften. Sie folgte der Logik der „Ordnungspolitik der sichtbaren Hand“ (Werner Abelshauser). Solche Politiken und Allianzen konzentrierten sich auf die schrittweise Entwicklung von Innovationen sowie auf die Verbreitung neuen Wissens und neuer Praktiken durch öffentliche Forschungs- und Bildungseinrichtungen. Und sie funktionierte bis in die 1990er Jahre hinein spektakulär gut. Wie aus Abbildung 1 hervorgeht, werden private Investitionen in Forschung und Entwicklung (FuE) in Deutschland – mit Ausnahme Ostdeutschlands – von einer Vielzahl öffentlicher Forschungseinrichtungen ergänzt.[1] Dies deutet darauf hin, dass der deutsche Staat

1 Fraunhofer-Gesellschaft, Max-Planck-Gesellschaft, Helmholtz-Gemeinschaft, Deutsches Zentrum für Luft- und Raumfahrt, Leibniz-Gemeinschaft.

Abbildung 1
**Ausgaben für private Forschung und Entwicklung und Zahl der öffentlichen Forschungsinstitute**
NUTS2-Regionen

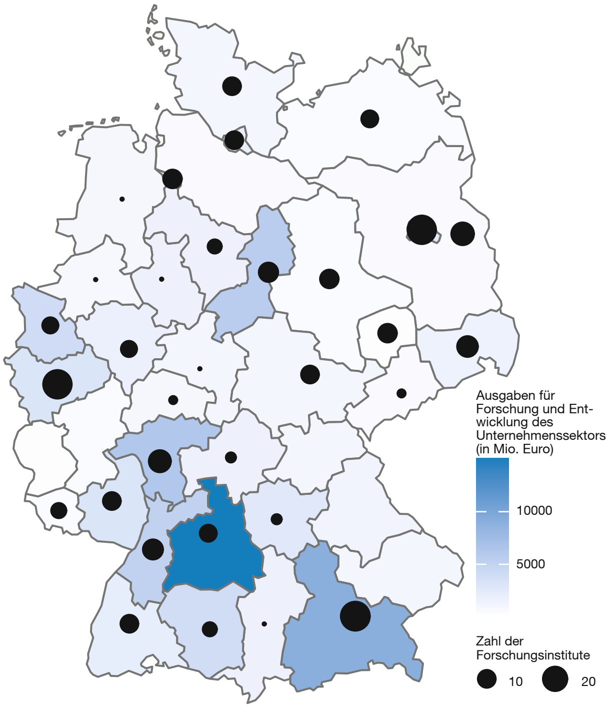

Quellen: Eurostat, Fraunhofer-Gesellschaft, Max-Planck-Gesellschaft, Helmholtz-Gemeinschaft, Deutsches Zentrum für Luft- und Raumfahrt, Leibniz-Gemeinschaft, eigene Berechnungen.

Abbildung 2
**Gini-Index nach Markteinkommen und verfügbarem Einkommen, Deutschland und Korea**

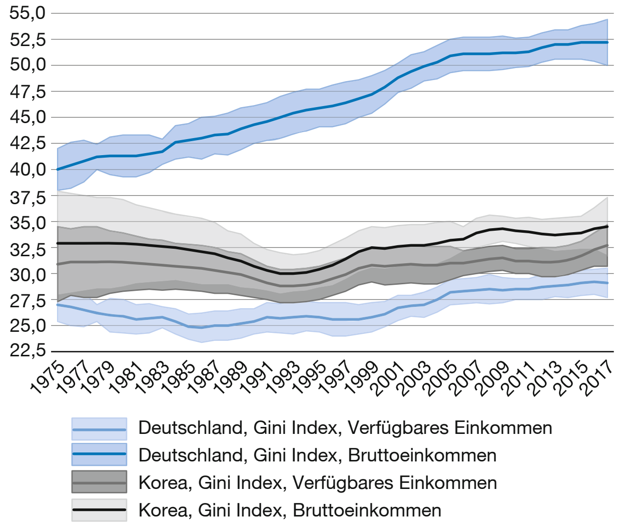

Quelle: Solt (2020).

bisher nicht in der Lage war, die Erfolge der Nachkriegszeit nach der Wiedervereinigung in Ostdeutschland zu wiederholen (Reinert, 2014).

Das wirtschaftspolitische Modell der Nachkriegszeit beruhte auch auf einer weitreichenden Koordinierung der Steuer-, Wohlfahrts- und Wirtschaftspolitik, die es großen Teilen der Gesellschaft ermöglichte, die Früchte des Wachstums zu genießen. Dieser Pakt gilt nicht mehr. In den letzten Jahrzehnten ist die Ungleichheit zu einer der größten Herausforderungen für das Land geworden. Wie Abbildung 2 zeigt, haben Deutschland und Südkorea ähnliche Verteilungskoeffizienten der verfügbaren Einkommen, doch „hinter dieser bemerkenswerten Ähnlichkeit verbirgt sich ein großer Unterschied in der Art und Weise, wie die Einkommen entstehen, wobei der Weg Deutschlands weitaus verschlungener ist als der Koreas: Um zu einem Gini-Koeffizienten des verfügbaren Einkommens von etwa 30 zu gelangen, muss Deutschland den Brutto-Gini-Koeffizienten um 44 % verringern, während Korea nur 9 % benötigt“ (Palma, 2019). Die Verteilungsintensität des deutschen Systems war eines der Schlüsselmerkmale des ordoliberalen Konsenses. Diese Verbindung zwischen industrieller Entwicklung und „Entwicklungsgesellschaft“ (Weiss, 1998) ist nun aufgehoben.

In Reaktion auf COVID-19 wurden jedoch Diskussionen über die deutsche Wirtschaftspolitik geführt, die einige Grundlagen neu überdenken. Deutschland hat nach der Pandemie mutige politische Maßnahmen zur Umgestaltung seiner Wirtschaft ergriffen. Viele Länder gewähren Unternehmen bedingungslose Kredite, wobei Deutschland auch in Erwägung zieht, sich an notleidenden Unternehmen zu beteiligen, eine Idee, die bei führenden deutschen Wirtschaftswissenschaftlern breite Unterstützung zu finden scheint. Während die deutsche Reaktion auf die globale Finanzkrise in den 2010er Jahren weitgehend von Sparmaßnahmen und der Unterstützung bestehender Industriepraktiken wie der Abwrackprämie für Altautos geprägt war, hat die Regierung diesmal in ihren am 3. Juni 2020 veröffentlichten Unterstützungsmaßnahmen einen mutigeren nachhaltigen Ansatz gewählt. Seit Beginn der Pandemie im Jahr 2020 hat die deutsche Regierung 50 Mrd. Euro zur Unterstützung verschiedener Energiearten durch 18 neue oder geänderte Maßnahmen zugesagt. Abbildung 3 zeigt, dass Deutschland im Vergleich zu anderen G20-Ländern mit insgesamt 24 Mrd. Euro den größten Betrag für saubere Energie zugesagt hat.

Und obwohl es Anzeichen für einen sich wandelnden wirtschaftlichen Konsens gibt, scheint es in der kollek-

Abbildung 3
**COVID-19-Konjunkturpakete aus einer Klima- und Energieperspektive**
in Mrd. US-$

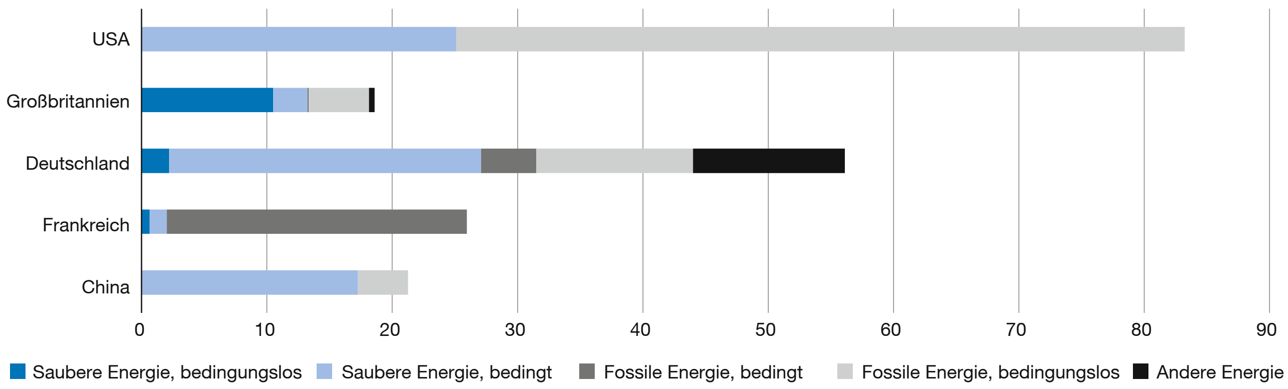

Quelle: EnergyPolicyTracker, https://www.energypolicytracker.org/region/g20/, eigene Berechnungen.

tiven politischen Denkweise eher darum zu gehen, zu den führenden Volkswirtschaften aufzuschließen, anstatt eine führende Volkswirtschaft zu sein. Tatsächlich fällt Deutschland in mancher Hinsicht zurück. Sein Anteil an Patenten in Zukunftstechnologien sinkt (Bertelsmann, 2020). Die viel gepriesene Energiewende scheint in einer Vielzahl von Förderprogrammen und mangelnder politischer Koordination steckenzubleiben (Gatzen et al., 2019).

Eine Industriestrategie, die es ermöglicht, große Herausforderungen wie die Klimakatastrophe zu bewältigen, erfordert die Wiederbelebung sowohl privater als auch öffentlicher Investitionen, Innovation und Zusammenarbeit. Es geht nicht um „mehr Staat" oder „weniger Staat", sondern um eine andere Art von Staat (Mazzucato, 2016).[2] Einer, der in der Lage ist, als Investor zu agieren, neue Arten von Wachstum zu katalysieren und auf diese Weise Investitionen und Innovationen des privaten Sektors anzuregen, was Erwartungen an zukünftige Wachstumsbereiche weckt. Dies erfordert eine neue Form der Zusammenarbeit zwischen Staat und Wirtschaft – es geht eher um die Wahl der „Willigen" als der Gewinner.

2 Diese Strategie erfordert langfristige strategische Investitionen und eine Politik, die darauf abzielt, Märkte zu schaffen und zu gestalten, anstatt nur Märkte oder Systeme zu „reparieren". Die Schaffung von Märkten charakterisiert auftragsorientierte Investitionen, die dazu geführt haben, einen Menschen auf den Mond zu bringen, und die heute grüne Innovationen anregen. Auftragsorientierte Innovationen verlangen von den öffentlichen Stellen nicht nur, dem privaten Sektor Risiken abzunehmen, sondern auch die direkte Schaffung neuer technologischer Möglichkeiten und Marktlandschaften anzuführen. Dieses Papier befasst sich mit vier Schlüsselfragen, die sich aus einem marktschaffenden Rahmen für die Politik ergeben.

Wie Carlota Perez (2003) zeigt, dass es für das gegenwärtige durch die Informations- und Kommunikationstechnik (IKT) gesteuerte Industrieparadigma von entscheidender Bedeutung ist, zu verstehen, dass unser Lebensstil ebenso wichtig ist wie die Modernisierung und Umgestaltung der Industrie. Ein intelligenter grüner Lebensstil, so argumentiert sie, „als Richtung für Innovationen könnte der beste Weg sein, um eine erfolgreiche Einführung des IKT-Zeitalters zu erreichen ... Als Richtung für Innovationen und Investitionen umfasst er eine Veränderung der Lebens- und Konsumgewohnheiten, die zu einer Verschiebung der Materialien und des Produktdesigns führt, wie in diesem Fall den zunehmenden Ersatz von materiellen Gütern durch Dienstleistungen". Dies bedeutet, dass neue Industriestrategien sowohl den Lebensstil als auch die industrielle Transformation in Angriff nehmen müssen.

Wir sind der Auffassung, dass politische Entscheidungsträger durch klar definierte Ziele oder genauer gesagt „Missionen", die auf die Lösung wichtiger gesellschaftlicher Herausforderungen ausgerichtet sind, die Möglichkeit haben, die Richtung des Wachstums zu bestimmen, indem sie strategische Investitionen tätigen, Maßnahmen über viele verschiedene Sektoren hinweg koordinieren und neue Industrielandschaften fördern, die der Privatsektor weiterentwickeln kann (Mazzucato, 2018). Das Ergebnis wäre eine Steigerung des sektorübergreifenden Lernens und der makroökonomischen Stabilität. Bei einem solchen herausfordernden und marktgestaltenden Ansatz geht es nicht um eine Planung von oben nach unten durch einen übermächtigen Staat; es geht darum, eine Richtung für das Wachstum vorzugeben, die Erwartungen der Unternehmen an künftige Wachstums-

Abbildung 4

**Das KfW-Inlandsvolumen und die Investitionen der KfW in den Umwelt- und Klimaschutz**

in Mrd. Euro

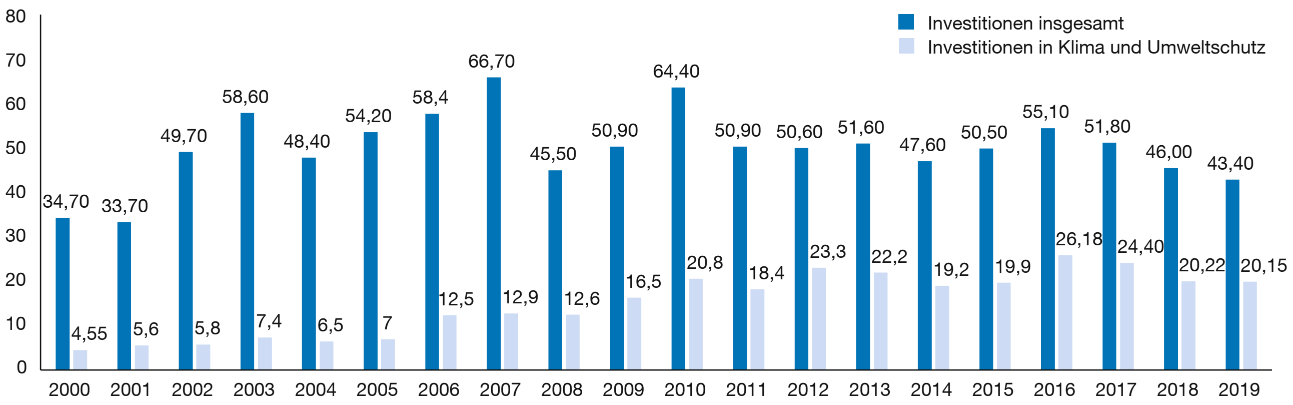

Quelle: KfW, Geschäftsberichte (bis 2014) und Förderreporte (ab 2015), eigene Berechnungen.

bereiche zu erhöhen und eine Aktivität – als Selbstentdeckung der Unternehmen – zu katalysieren, die sonst nicht stattfinden würde. Es geht weder darum, die Risiken zu verringern und das Spielfeld zu nivellieren, noch darum, wettbewerbsfähigere Sektoren weniger zu unterstützen, da der Markt es nicht immer am besten weiß, sondern darum, das Spielfeld in Richtung der gewünschten gesellschaftlichen Ziele zu kippen, wie z. B. die Verhinderung des klimatischen Notstands.

Die deutsche Industrie- und Innovationspolitik hat sich langsam auf einen solchen missionsorientierten Ansatz zubewegt. Eine zentrale Säule ist dabei die größte deutsche Landesförderbank Kreditanstalt für Wiederaufbau (KfW), die Industriepolitik durch die Vergabe von Krediten an Schwerpunktbereiche betreibt. Gemessen an der Größe ihrer Aktiva ist die KfW einflussreicher als ihre chinesischen und brasilianischen Pendants CDB und BNDES (Mazzucato und Penna, 2016). Eine der größten Erfolgsgeschichten der KfW und des missionsorientierten Ansatzes in Deutschland ist der Übergang des Energiesystems im Rahmen der Energiewende. Statt nur den politischen Rahmen für den Markt zu schaffen, um eine stärkere Nutzung erneuerbarer Energien zu erreichen, hat sich der deutsche Staat auf die kühne Mission der Energiewende eingelassen. Durch den Einsatz von Einspeisetarifen und die Bereitstellung subventionierter langfristiger Finanzierungen über die KfW hat Deutschland seit Ende der 1990er Jahre eine Binnennachfrage zur Förderung der Nutzung erneuerbarer Energien geschaffen. Abbildung 4 zeigt den dramatischen Anstieg der Investitionen der KfW in den Umwelt- und Klimaschutz seit dem Jahr 2000.

Die Energiewende schuf wohl einen globalen Markt für Sonnenkollektoren (Fagerberg, 2018) und führte zu mehr Innovation und Investitionen in den grünen Übergang. Im Jahr 2014 stellten deutsche Unternehmen ca. 50 % aller europäischen Unternehmensinvestitionen in energiebezogene FuE bereit (Umweltbundesamt, 2019).

Die Energiewende kann als ein erfolgreicher Fall beschrieben werden, bei dem missions- und diffusionsorientierte Elemente der Innovationspolitik kombiniert wurden. Die Zusammenarbeit zwischen verschiedenen Ministerien und Institutionen wie BMWi, BMF und KfW bietet einen verlässlichen institutionellen Rahmen für eine missionsorientierte Innovationspolitik.

Im Jahr 2018 hat die Bundesregierung mit der Hightech-Strategie 2025 eine rahmenpolitische Agenda eingeführt, die eine übergreifende Koordinierungsstrategie für die deutsche Wissenschafts-, Technologie- und Innovationspolitik darstellt. Ihr Ziel ist es, gemeinsam mit den Ländern und der Wirtschaft die FuE-Ausgaben auf 3,5 % des Bruttoinlandsprodukts zu steigern. Dabei wird versucht, die deutschen Forschungseinrichtungen und die Forschungsförderung vor sozioökonomische Herausforderungen zu stellen. Die Strategie umfasst 12 Missionen, fungiert aber vor allem als ressortübergreifendes Koordinationsinstrument, um die bestehende, weitgehend diffusionsorientierte Forschungs- und Industrielandschaft und die sozioökonomischen Herausforderungen besser aufeinander abzustimmen.

Allerdings gibt es in der deutschen Industrielandschaft erhebliche unausgelastete Ressourcen. Ein Thema, das in der wissenschaftlichen Debatte zunehmend Beachtung findet, ist die Rolle von Zentralbanken und Finanzregulatoren bei der Bewältigung klimabedingter Finanzrisiken (Campiglio et al., 2018). Seit der globalen Finanzkrise von 2008 haben die Zentralbanken zunehmend ein breiteres Spektrum „unkonventioneller" Maßnahmen eingesetzt, darunter quantitative Lockerung und verschiedene andere kurz- und längerfristige Liquiditätsprogramme zur Stimulierung der Wirtschaft. Die Ausweitung der Zentralbankinterventionen in die Märkte bietet eine ausgezeichnete Gelegenheit, die Finanzströme strategischer auf umweltfreundlichere, kohlenstofffreie Alternativen umzulenken. Trotz dieses großen Potenzials deuten Untersuchungen darauf hin, dass die Käufe von Unternehmensanleihen durch die Europäische Zentralbank (EZB) die Investitionsentscheidungen der Finanzmärkte widerspiegeln und dadurch bisher vor allem große kohlenstoffintensive Unternehmen begünstigt haben (Jourdan und Kalinowski, 2019; Matikainen, Campiglio und Zenghelis, 2017). Um diese unerwünschte Konsequenz zu verhindern, sollten die Zentralbanken entweder die Käufe im Rahmen der quantitativen Lockerung neu kalibrieren, um kohlenstoffintensive Finanzanlagen auszuschließen, oder parallel dazu ein grünes quantitatives Lockerungsprogramm durchführen, um den Effekt abzuschwächen.

In der Eurozone führen die Zentralbanken Belgiens, Finnlands, Frankreichs, Deutschlands, Italiens und Spaniens im Auftrag der EZB Anleihekäufe durch. Dies wird von jeder Nationalbank in ihrem jeweiligen Tätigkeitsbereich durchgeführt. Das heißt, die Deutsche Bundesbank kauft beispielsweise Anleihen von Unternehmen in Deutschland und den Niederlanden, die Banque de France kauft Anleihen von französischen Unternehmen und so weiter. Während die Käufe in die Bilanz der jeweiligen Nationalbank eingehen, obliegt die Koordination der EZB. Infolgedessen werden etwaige finanzielle Gewinne oder Verluste nach dem Kapitalschlüssel der EZB auf alle Nationalbanken des Eurosystems verteilt. Untersuchungen haben ergeben, dass der Großteil der Anleihekäufe von Unternehmen getätigt wird, die in den kohlenstoffintensivsten Sektoren tätig sind: Gewinnung und Verteilung fossiler Energieträger, Automobilherstellung und -ausrüstung sowie Versorgungsunternehmen (Jourdan und Kalinowski, 2019).

Da die Bundesbank das Programm zur quantitativen Lockerung jedoch im Auftrag der EZB durchführt, ist die faktische Macht der Bundesbank relativ gering. Dennoch ist der Einfluss Deutschlands auf die EZB erheblich. Zumindest erscheint es angemessen, dass die Bundesbank ihre Kriterien für den Erwerb von Vermögenswerten aktiv überprüft, damit klimabedingte Finanzrisiken angemessen in ihre Programme zum Erwerb von Sicherheiten und Vermögenswerten integriert sind (Chenet, Ryan-Collins und van Lerven, 2019).

Eine weitere nicht ausgeschöpfte Finanzierungsquelle, die sowohl Lebensstile als auch die industrielle Produktion verändert, ist die Beschaffung. Die öffentliche Hand in der EU gibt jedes Jahr rund 2 Billionen Euro für diese Anschaffungen aus, was 14 % des BIP entspricht; allein Deutschland gibt bis zu 500 Mrd. Euro für Beschaffungen aus. Angesichts dieser enormen Kaufkraft birgt eine grüne oder nachhaltige Beschaffung ein großes Potenzial zur Dekarbonisierung der Wirtschaft. Im Gegensatz zu Emissionshandelssystemen, deren Preise derzeit zu niedrig sind, um einen kohlenstoffarmen Übergang zu bewirken, bietet die grüne Beschaffung einen bedeutenden und unmittelbaren Weg in die Zukunft (Chiappinelli und Zipperer, 2017). Als positiver Spillover-Effekt hat die grüne Beschaffung das Potenzial, die Entwicklung von Leitmärkten für klimafreundliche Technologien anzustoßen und Anreize für grüne Innovationen zu schaffen.

Das deutsche Vergaberecht ermöglicht es der öffentlichen Hand, strategische Ziele, wie z.B. Umweltanforderungen, in die Vergabekriterien des Bieterverfahrens einzubeziehen. Wie Untersuchungen zeigen, enthielten jedoch nur 2,4 % aller öffentlichen Aufträge, die 2015 in Deutschland vergeben wurden, Umweltkriterien für das öffentliche Beschaffungswesen. Während Deutschland auf Bundesebene ehrgeizige Ziele für die Dekarbonisierung formuliert hat, scheint dies auf der Ebene der Bundesländer und Kommunen nicht erreicht zu werden, wo eine Reihe von Herausforderungen und Hindernisse für eine umweltfreundliche Beschaffungspraxis bestehen.

Die deutsche Politik sollte die einzigartige Gelegenheit, die durch die globale Pandemie geschaffen wurde, für weitere institutionelle und politische Innovationen für gezieltere Investitionen sowohl des öffentlichen als auch des privaten Sektors nutzen. Um dies zu erreichen, braucht Deutschland ein neues wirtschaftspolitisches Modell, das ausdrücklich darauf abzielt, die Märkte für ein nachhaltigeres und gerechteres Wachstum zu gestalten. Ein marktgestaltender Politikrahmen setzt auf Komplementaritäten und Ausgewogenheit zwischen diffusionsorientierten Politiken und dem Fokus auf die Lösung spezifischer sozioökonomischer Herausforderungen (Missionen). Er muss den Innovationsauftrag auch auf die Finanzregulierung, das Beschaffungswesen und allgemeinere wirtschaftspolitische Institutionen wie Zentralbanken übertragen.

Im Falle Deutschlands bedeutet die Anwendung eines marktgestaltenden politischen Rahmens, dass es auf seinen bestehenden Stärken bei der Wissensverbreitung durch öffentliche und private Akteure aufbaut und neu entstehende auftragsorientierte Politiken und Institutionen stärkt, wie es teilweise bei seinen Reaktionen auf die COVID-19-Pandemie geschehen ist. Deutschland verfügt über ein enormes Potenzial, seinen Finanzsektor und seine öffentlichen Finanzen (insbesondere das öffentliche Beschaffungswesen) auf die in der Hightech-Strategie 2025 festgelegten Missionen auszurichten. Wir meinen, dass dies zusätzlich zu einer besseren Koordinierung der bestehenden Industrie- und Innovationspolitik die Bereiche sind, in denen Deutschland robustere dynamische Fähigkeiten in den öffentlichen Organisationen aufbauen muss. Insbesondere besteht ein wesentlicher Unterschied zwischen Finanzierungsarten, die Investitionen in die Realwirtschaft begünstigen, und spekulativen Finanzierungen, die risikoarmen, kurzfristigen Kapitalgewinnen durch den Handel mit vorhandenen Vermögenswerten Vorrang einräumen. Da Innovation ein kollektiver, unsicherer und kumulativer Prozess ist, erfordert sie eine langfristige, engagierte und geduldige Finanzierung. Dies ist die Art von Finanzierung, die in den heutigen Finanzsektoren oft fehlt, und sie ist im privaten Bereich immer schwerer zu finden.

Grundsätzlich sollten sich die deutsche Innovations-, Industrie-, Finanz- und Beschaffungspolitik ergänzen und über unabhängige Initiativen und diskrete Ansätze hinausgehen. Die Märkte werden nicht von sich aus eine grüne und integrative Richtung für Innovationen finden. Es gibt noch keinen vorgefertigten Weg, der multidirektionale, experimentelle, grüne und integrative Innovationen rentabel macht. Nur wenn es eine stabile und konsistente Richtung für Investitionen gibt, werden Regulierungen und Innovationen auf einem grünen und integrativen Pfad zusammenlaufen. Der Übergang muss durch eine langfristige, geduldige Finanzierung gefördert werden, die bereit ist, Risiken einzugehen, und in der Lage ist, andere Investoren zu mobilisieren und zu drängen. Um zu verhindern, dass die Innovation weiterhin auf einem kohlenstoffreichen Pfad verlaufen, der mit zunehmender Ungleichheit gepflastert ist, und um stagnierenden Innovationslandschaften den Rücken zu kehren, muss die öffentliche Politik sicherstellen, dass Investitionen in kohlenstoffarme und integrative Innovationen belohnt werden. Dies kann erreicht werden, indem die gesamte und koordinierte Palette staatlicher Instrumente genutzt wird, um „die Willigen" auszuwählen: jene Organisationen, die bereit sind, die schwierigen Anstrengungen auf sich zu nehmen, die für einen grünen und integrativen Übergang erforderlich sind. Die Regierung kann diesen Prozess nicht auf Mikro-Ebene managen, da dies die Innovation ersticken würde. Aber sie kann eine klare Richtung vorgeben, die ersten risikoreichen Investitionen tätigen, die später private Akteure anziehen, und diejenigen belohnen, die bereit sind, zu investieren und innovativ zu sein.


Prof. Rainer Kattel, Ph.D., ist stellvertretender Direktor und Professor am Institute for Innovation and Public Purpose (IIPP) des University College London.

Prof. Mariana Mazzucato, Ph.D., ist Direktorin und Professorin am Institute for Innovation and Public Purpose (IIPP) des University College London.

Keno Haverkamp ist Research Assistant am University College London.

Josh Ryan-Collins, Ph.D., ist Head of Finance and Macroeconomics und Senior Research Fellow am Institute for Innovation and Public Purpose (IIPP) des University College in London.


Literatur

Bertelsmann Stiftung (2020), Weltklassepatente in Zukunftstechnologien, https://www.bertelsmann-stiftung.de/de/publikationen/publikation/did/weltklassepatente-in-zukunftstechnologien (8. Oktober 2020).
Campiglio E Climate Change Challenges for Central Banks and Financial Regulators Nature Climate Change 2018 8 6 462 468 10.1038/s41558-018-0175-0
Chenet H Ryan-Collins und J van Lerven F Climate-Related Financial Policy in a World of Radical Uncertainty, UCL Institute for Innovation and Public Purpose (IIPP) Working Paper Series 2019 2019–13 23
Chiappinelli O Zipperer V Using Public Procurement as a Decarbonisation Policy: A Look at Germany DIW Economic Bulletin 2017 7 49 523 532
Fagerberg, J. (2018), Mission (Im)Possible? The Role of Innovation (and Innovation Policy) in Supporting Structural Change & Sustainability Transitions, Working Papers on Innovation Studies, (Centre for Technology, Innovation and Culture, University of Oslo), https://ideas.repec.org/p/tik/inowpp/20180216.html (8. Oktober 2020).
Gatzen, C. et al. (2019), Technologische Innovationen und neue Geschäftsmodelle für die Energiewende — Die Rolle der deutschen F&I Politik: Studie im Auftrag der unabhängigen Expertenkommission Forschung und Innovation (EFI), Research Report (Studien zum deutschen Innovationssystem, 2019), https://www.econstor.eu/handle/10419/194281 (8. Oktober 2020).
Jourdan, S. und W. Kalinowski (2019), Aligning Monetary Policy with EU’s Climate Targets, Veblen Institute for Economic Reforms and Positive Money Europe.
Matikainen, S., E. Campiglio und D. Zenghelis (2017), The Climate Impact of Quantitative Easing, Policy Paper.
Mazzucato M From Market Fixing to Market-Creating: A New Framework for Innovation Policy’ Industry and Innovation 2016 23 2 140 156 10.1080/13662716.2016.1146124
Mazzucato, M. (2018), Mission-Oriented Research & Innovation in the European Union: A Problem-Solving Approach to Fuel Innovation-Led Growth, Website (Publications Office of the European Union, 21. Februar 2018), http://op.europa.eu/en/publication-detail/-/publication/5b2811d1-16be-11e8-9253-01aa75ed71a1/language-en (8. Oktober 2020).
Mazzucato M Penna C C R Beyond Market Failures: The Market Creating and Shaping Roles of State Investment Banks Journal of Economic Policy Reform 2016 19 4 305 326 10.1080/17487870.2016.1216416
Palma J G Behind the Seven Veils of Inequality. What If It’s All about the Struggle within Just One Half of the Population over Just One Half of the National Income? Development and Change 2019 50 5 1133 1213 10.1111/dech.12505
Perez, C. (2003), Technological Revolutions and Financial Capital: The Dynamics of Bubbles and Golden Ages, Edward Elgar Publishing.
Reinert, E. S. (2014), Warum Manche Länder Reich Und Andere Arm Sind: Wie Der Westen Seine Geschichte Ignoriert Und Deshalb Seine Wirtschaftsmacht Verliert, Schäffer-Poeschel, https://www.amazon.co.uk/dp/B07V3T2JNH/ref=dp-kindle-redirect?_encoding=UTF8&btkr=1 (8. Oktober 2020).
Solt F Measuring Income Inequality Across Countries and Over Time: The Standardized World Income Inequality Database Social Science Quarterly 2020 101 3 1183 1199 10.1111/ssqu.12795
Umweltbundesamt (2019), Innovationsmotor Umweltschutz: Forschung und Patente in Deutschland und im nationalen und internationalen Vergleich, https://www.umweltbundesamt.de/sites/default/files/medien/1410/publikationen/2019-12-05_uib_06-2019_innovationsmotor-umweltschutz-2019.pdf (8. Oktober 2020).
Weiss, L. (1998), The Myth of the Powerless State, 1. Aufl., Cornell University Press.
